# Development and evaluation of a rapid and sensitive RPA assay for specific detection of *Vibrio parahaemolyticus* in seafood

**DOI:** 10.1186/s12866-019-1562-z

**Published:** 2019-08-13

**Authors:** Yunyun Geng, Ke Tan, Libing Liu, Xiao Xia Sun, Baohua Zhao, Jianchang Wang

**Affiliations:** 1 Department of Pharmacology, Hebei University of Chinese Medicine, No.326 South Xinshi Road, Shijiazhuang, 050091 Hebei China; 2 College of Life Sciences, Hebei Normal University, No.20, Road E. 2nd Ring South, Yuhua District, Shijiazhuang, Hebei Province 050024 People’s Republic of China; 3 Center of Inspection and Quarantine, Hebei Entry-Exit Inspection and Quarantine Bureau, No.318 Hepingxilu Road, Shijiazhuang, 050024 Hebei China; 4 Hebei Academy of Inspection and Quarantine Science and Technology, No.318 Hepingxilu Road, Shijiazhuang, Hebei Province 050051 People’s Republic of China

**Keywords:** *Vibrio parahaemolyticus*, *gyrB*, Real-time RPA, Molecular detection

## Abstract

**Background:**

*Vibrio parahaemolyticus (V. parahaemolyticus)* is a leading cause of food poisoning and is of great importance to public health due to the frequency and seriousness of the diseases. The simple, timely and efficient detection of this pathogen is a major concern worldwide. In this study, we established a simple and rapid method based on recombinase polymerase amplification (RPA) for the determination of *V. parahaemolyticus*. According to the *gyrB* gene sequences of *V. parahaemolyticus* available in GenBank, specific primers and an exo probe were designed for establishing real-time recombinase polymerase amplification (real-time RPA).

**Results:**

The real-time RPA reaction was performed successfully at 38 °C, and results were obtained within 20 min. The method only detected *V. parahaemolyticus* and did not show cross-reaction with other bacteria, exhibiting a high level of specificity. The study showed that the detection limit (LOD) of real-time RPA was 1.02 × 10^2^ copies/reaction. For artificially contaminated samples with different bacteria concentrations, *V. parahaemolyticus* could be detected within 5–12 min by real-time RPA in oyster sauce, codfish and sleeve-fish at concentrations as low as 4 CFU/25 g, 1 CFU/25 g and 7 CFU/25 g, respectively, after enrichment for 6 h, but were detected in a minimum of 35 min by real-time PCR (Ct values between 27 and 32)*.*

**Conclusion:**

This study describes a simple, rapid, and reliable method for the detection of *V. parahaemolyticus*, which could potentially be applied in the research laboratory and disease diagnosis.

## Background

*Vibrio parahaemolyticus* (*V. parahaemolyticus*) is a gram-negative, halophilic, rod-shaped bacterium belonging to the family *Vibrionaceae*. It is naturally present in brackish coastal environments and is frequently isolated from a variety of seafood [1, 2]. *V. parahaemolyticus* is a major seafood-borne pathogen that causes gastrointestinal disorders due to ingestion of raw or undercooked seafood contaminated with this pathogen [3, 4]. In recent years, outbreaks of *V. parahaemolyticus* infections have been a significant public health concern in many countries. Additionally, it should be noted that the number of *V. parahaemolyticus* infections has increased, and their reach has widened globally during recent years [5–7]. *V. parahaemolyticus-*infected persons are characterized by an acute gastroenteritis disorder with clinical signs of diarrhea, headache, and vomiting [4, 8, 9]. Low immunity populations who become infected with *V. parahaemolyticus* may develop septicemia in severe cases [10]. Controlling the amount of *V. parahaemolyticus* in seafood is an effective way to prevent infection by this pathogen; therefore, it is important to determine the levels of *V. parahaemolyticus* in seafood [11].

Early and rapid diagnosis is crucial for the management of *V. parahaemolyticus* infection. Different diagnostic methods for *V. parahaemolyticus* infection have been reported. Conventional culturing and immunological methods are common techniques used for *V. parahaemolyticus* detection [2, 9, 12, 13]. However, they are time-consuming and take a few days to provide a confirmed result after numerous analytical steps [12–14]. Advancements in biotechnology have led to the development of a number of gene amplification-based molecular detection technologies for *V. parahaemolyticus*, with varying degrees of sensitivity and specificity. These include polymerase chain reaction (PCR) [1], real-time PCR [11, 15], loop-mediated isothermal amplification (LAMP) [16, 17], and cross-priming amplification (CPA) [18, 19]. PCR has been widely employed for the rapid detection of *V. parahaemolyticus.* However, it is impractical for on-site application due to the expensive thermal cycling instruments required and time-consuming operation. Recently, a LAMP method was reported for rapid and sensitive detection of *V. parahaemolyticus,* but the reaction time and temperature were approximately 1 h and 65 °C [16]. A simple, rapid, accurate and user-friendly platform is still needed for the early point-of-need (POD) detection of *V. parahaemolyticus* infection.

Recombinase polymerase amplification (RPA), a novel isothermal gene amplification technique, has been demonstrated to be a simple, rapid, specific, sensitive and cost-effective molecular assay to identify diverse pathogens [20–25]. The RPA process relies on three core enzymes: a recombinase, a single-stranded DNA-binding protein (SSB) and a strand-displacing polymerase. The recombinase is capable of pairing the primer with the homologous sequence in the target DNA [25]. SSB binds to the strand of DNA displaced by the primer and stabilizes the D-loop that has formed to prevent the dissociation of primers. Finally, the strand-displacing polymerase adds bases to the 3′ end of the primer and primer extension occurs. When opposing primers are used, exponential amplification of the target sequence with RPA can be achieved in 20 min or less. This study describes the development and evaluation of a real-time RPA method that utilizes the fluorescent TwistAmp1 exo probe and portable instrumentation for the simple and rapid detection of *V. parahaemolyticus.*

## Methods

### Bacterial strains and DNA extraction

A total of 5 *V. parahaemolyticus* strains, 5 other *Vibrio* species and 22 other bacterial strains were used to determine the specificity of the real-time RPA (Table 1). These strains were stored in our lab. Stock cultures were stored at − 80 °C in 0.8 mL of Nutrient broth (Beijing Land Bridge Technology Co., Ltd., Beijing, China) and 0.2 mL of 80% glycerol. The DNA templates were extracted with the TIANamp Bacteria DNA Kit (Tiangen, Beijing, China). All DNA templates were stored at − 20 °C until assayed.

**Table 1:** Bacterial strains used in the specificity test

Stains	Origin	Real-time RPA	Real-time PCR
*Vibrio parahaemolyticus*	CICC 21617	+	+
*Vibrio parahaemolyticus*	ATCC 17802	+	+
*Vibrio parahaemolyticus*	Isolated in our lab	+	+
*Vibrio parahaemolyticus*	Isolated in our lab	+	+
*Vibrio parahaemolyticus*	Isolated in our lab	+	+
*Vibrio mimicus*	*CICC10474*	–	–
*Vibrio cholera*	*ATCC51394*	–	–
*Vibrio anguillarum*	*CICC10475*	–	–
*Vibrio alginolyticus*	*CICC21664*	–	–
*Vibrio vulnificus*	CICC21615	–	–
*Bacillus cereus*	ATCC 11778	–	–
*Bacillus cereus*	CICC 10648	–	–
*Campylobacter jejuni*	ATCC 33291	–	–
*Citrobacter freundii*	ATCC 10787	–	–
*Cronobacter sakazakii*	ATCC 29544	–	–
*Cronobacter sakazakii*	ATCC 21548	–	–
*Escherichia coli O157:H7*	CICC 21530	–	–
*Escherichia coli*	CMCC 44102	–	–
*Enterococcus faecalis*	ATCC 29212	–	–
*Klebsiella pneumoniae*	ATCC 4352	–	–
*Listeria monocytogenes*	ATCC 19114	–	–
*Proteus mirabilis*	ATCC 29906	–	–
*Providencia*	ATCC 29944	–	–
*Pseudomonas aeruginosa*	Isolated by lab	–	–
*Salmonella typhimurium*	CICC 22956	–	–
*Serratia marcescens*	ATCC 14756	–	–
*Shigella sonnei*	CICC 21679	–	–
*Shigella sonnei*	ATCC 25931	–	–
*Shigella flexneri*	CICC 21678	–	–
*Staphylococcus aureus*	ATCC 6538	–	–
*Staphylococcus aureus*	ATCC 25923	–	–
*Yersinia enterocolitica*	CICC 21609	–	–

+, positive result; −, negative result

### RPA primers and probe

Nucleotide sequence data for *V. parahaemolyticus* strains from GenBank were aligned to identify conserved regions. According to the reference sequences of different *V. parahaemolyticus* genotypes (accession numbers: AM235735, DQ316918, FM202616, and EU051591), three pairs of primers targeting the conserved region of *gyrB* were designed. The real-time RPA primers and probes were selected by testing the combination that yielded the highest sensitivity (Table 2). Primers and exo probes were synthesized by Sangon (Sangon, Shanghai, China).

**Table 2:** Primer and probe sequences for *V. parahaemolyticus* real-time PCR, RPA and real-time RPA assay

Method	Name	Sequence 5′-3’Amplication	size (bp)
Real-time RPA	RPA-FP	CGAAGAAAGCGAAAACGGCAACGTCAGGCGA	168
	RPA-RP	CAGATAATTTCTCACCCATCGCCGATTCAACC	
	exo Probe	GGTTTGACAGCCGTTGTTTCAGTAAAAGTGCC [FAM-dT]-THF-[BHQ-dT]TCCAAAATTCTCGAGCC	
Real-time PCR	PCR-FP	CGGTAGTAAACGCACTGTCAGAA	77
	PCR-RP	ACGGTAAGTTTGCGTGTGGAT	
	PCR-Probe	FAM- TGGTACTAACCATCCATCGTGGCGGTC -BHQ1	

### Real-time RPA reactions

Real-time RPA was carried out as describled previously [26, 27]. The Genie III scanner device (OptiGene Limited, West Sussex, UK) and TwistAmpTM exo kit (TwistDX, Cambridge, UK) was applied in the real-time RPA.

### Real-time PCR for *V. parahaemolyticus*

Real-time PCR was performed on the ABI 7500 instrument as described previously [27]. Premix Ex TaqTM (Takara Co., Ltd., Dalian, China) was used in the real-time PCR, and the reaction was performed as follows: 95 °C for 2 min, followed by 35 cycles of 95 °C for 10 s, 60 °C for 34 s. The sequences of the primers and probes used for real-time PCR are listed in Table 2. The reporter and fluorescence quencher was marked with 6-FAM (6-CarboxyFluorescein) BHQ1 (Black Hole Quencher 1) respectively.

### Specificity and analytical sensitivity analysis

One hundred nanograms of *V. parahaemolyticus* genomic DNA were used as the template for the specificity analysis of the real-time RPA assay. The assay was evaluated by a panel of pathogens considered to be important in food security (Table 1).

To evaluate the real-time RPA sensitivity, genomic DNA of *V. parahaemolyticus* was diluted in a 10-fold serial dilution to achieve DNA concentrations ranging from 1.0 × 10^6^ to 1.0 × 10^0^ copies/μL. One microliter of each DNA dilution was used as a template and amplified with the real-time RPA assay. Real-time RPA and PCR was tested using the standard DNA in 8 replicates. The threshold time was plotted against the molecules detected.

### Evaluation with artificially contaminated samples

*V. parahaemolyticus* was cultured in 9 mL of alkaline peptone water (APW) at 37 °C for 24 h, harvested by centrifugation at 4000 rpm for 20 min at 4 °C, and washed 3 times with PBS. The final pellet was then added to oyster sauce, codfish, or sleeve-fish using 10-fold serial dilutions to achieve different concentrations: 1–100 CFU/mL. The oyster sauce, codfish, and sleeve-fish, which were verified to be free of *V. parahaemolyticus* according to the National Standard GB 4789.7–2013 (People’s Republic of China, 2013), were purchased from a local supermarket. Twenty five grams of oyster sauce and 4, 25 and 80 CFU of *V. parahaemolyticus*; 25 g of codfish and 1, 10, and 42 CFU of *V. parahaemolyticus*; 25 g of sleeve-fish samples and 7, 10, and 56 CFU of *V. parahaemolyticus* were added into a sterile conical flask containing 225 mL APW (Land Bridge Technology, Beijing, China), mixed well to get homogenous samples, and then incubated for 6 or 8 h at 37 °C to increase the bacterial concentrations to detectable levels. Then, 1 mL aliquots were collected at each time point and centrifuged 10,000×g for 3 min at 4 °C. Then, the supernatant was carefully removed and the cell pellet was washed with PBS. After centrifugation, the cell pellet was resuspended and boiled for 10 min to release the DNA. The resulting liquid was used as the template for the subsequent RPA assay. Each experiment was repeated at least for three times, and similar results were obtained.

### Statistical methods

In order to determine the analytical sensitivity of the real-time PRA assay, a semi-log and probit regression was performed with Prism software 7.0 (Graphpad Software Inc., SanDiego, CA) and Statistical Product Service Solutions software (IBM, Armonk, NY, USA) as described previously [26].

## Results

### Performance of RPA

**Fig. 1:**
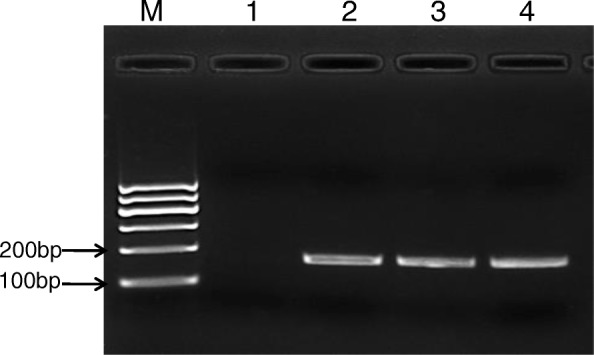
Optimization of RPA reaction time. Genomic DNA of *V. parahaemolyticus* was amplified with RPA for different lengths of time, a clear DNA band with the expected size (168 bp) could be visualized by agarose gel electrophoresis after a 20 min reaction. Semi-quantification of the DNA band density using image Bio-1D software of VILBER Fusion FX5 automatic gel imaging instrument (Vilber, Marne La Vallée, France) revealed that no significant difference was observed in the product yields of 30 min and 40 min reactions. M, DNA marker, lanes 1–4, DNA products from reactions incubated for 10 min, 20 min, 30 min and 40 min

The RPA reaction was performed as previously described using 100 ng of *V. parahaemolyticus* genomic DNA as template [22]. As shown in Fig. 1, an RPA product with the expected size (approximately 168 bp) was clearly visible after 20 min at 38 °C. Semi-quantification by measuring the DNA band density revealed that no significant difference was observed in the product yields of 20 min, 30 min and 40 min reactions (data not shown), which indicates that after 20 min, dNTPs or other components are completely consumed. Therefore, all RPA reactions were carried out for 20 min for the remainder of this study.

### Specificity and analytical sensitivity of real-time RPA

To assess the specificity of the real-time RPA assay under the conditions determined above, 5 *V. parahaemolyticus* strains and 27 other bacterial strains that frequently contaminate food were amplified by real-time RPA. The real-time RPA reaction was performed by using an exo probe and a portable, user-friendly tube scanner. As shown in Table 1, the 5 *V. parahaemolyticus* strains were detected while the other bacterial strains, including *Vibrio vulnificus, Vibrio alginolyticus, Vibrio mimicus, Vibrio anguillarum, Escherichia coli O157, S. aureus****,*** *Cronobater sakazakii, Campylobacter jejuni, Listeria monocytogenes, Shigella, Paratyphoid*, and *Bacillus cereus*, were not. Therefore, no cross-reaction was observed for the *V. parahaemolyticus* strain examined, and the real-time RPA assay was specific for the detection of *V. parahaemolyticus* bacteria.

**Fig. 2:**
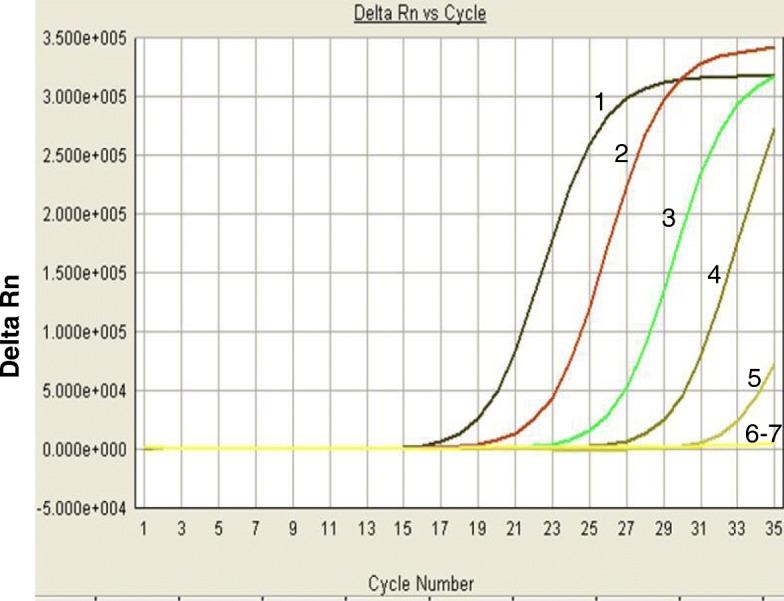
Sensitivity analysis of the real-time PCR assay. Different concentrations of *V. parahaemolyticus* DNA template (1.0 × 10^6^ to 1.0 × 10^0^ copies/reaction) were amplified by either real-time RPA or real-time PCR. As shown in this figure, the detection limit for both was 1.0 × 10^2^ copies/reaction. RPA assay is shown in panel A and real-time PCR is shown in panel B. The concentrations used as a template for reactions 1–7 were 1.0 × 10^6^, 1.0 × 10^5^, 1.0 × 10^4^, 1.0 × 10^3^, 1.0 × 10^2^, 1.0 × 10^1^ and 1.0 × 10^0^ copies/reaction. Shown in this figure is one representative plot out of five independent reactions for real-time RPA

**Fig. 3:**
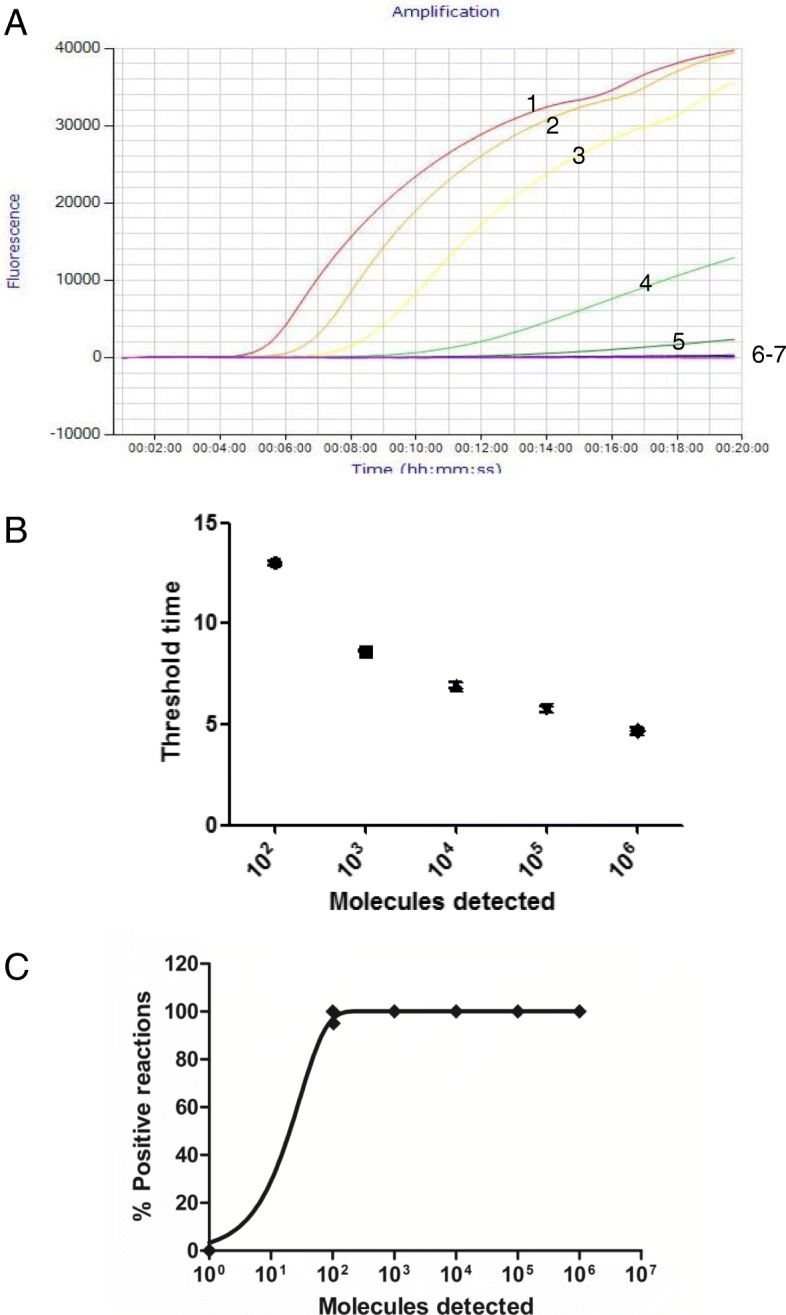
Performance of real-time RPA for detection of *V. parahaemolyticus*. **a**. Fluorescence development over time using a dilution range of 10^6^ to 10^0^ copies of the standard DNA as described above. **b**. Semi-logarithmic regression of the data collected from eight runs on the standard DNA using GraphPad Prism 7.0 (GraphPad Software Inc., San Diego, CA). The runtimes of real-time RPA were approximately 4.5 to 13 min for 10^6^ to 10^2^ copies. **c**. A probit regression analysis. The limit of detection of the real-time RPA was approximately 1.02 × 10^2^ copies/reaction in 95% of cases and indicated by a rhomboid

The analytical sensitivity of real-time RPA method was evaluated by Using*v. parahaemolyticus* genomic DNA as templates ranging from 1.0 × 10^6^ to 1.0 × 10^0^ copies/reaction. One microliter of each DNA dilution was amplified using both real-time RPA and real-time PCR. As shown in Fig. 3, the detection limit of real-time RPA was 10^2^ copies/reaction (panel A), while the detection limit of real-time PCR was also 10^2^ copies/reaction (Fig. 2). With the results of 8 complete molecular standard runs, a probit regression analysis revealed that the LOD of real-time RPA was 1.02 × 10^2^ copies/reaction in 95% of cases (Fig. 3c). A semi-log regression analysis was performed for the real-time RPA. The average reaction time from 8 runs of the DNA molecular was approximately 4.5–13 min for 10^7^–10^4^ copies (Fig. 3b). However, based on the real-time PCR Cq/Ct values, the real-time PCR LOD would be 1–2 log lower if the run cycles were increased to 40–45, and this would make real-time PCR more sensitive than real-time RPA assay.

### Evaluation with artificially contaminated samples

The diagnostic performance of the real-time RPA assay to detect *V. parahaemolyticus* in artificially contaminated food samples was compared to that of real-time PCR. Oyster sauce, codfish, and sleeve-fish are good substrates for *V. parahaemolyticus* growth and enterotoxin production and were contaminated by *V. parahaemolyticus* with different bacteria concentrations and enrichment times as shown in Tables 3, 4 and 5. The National Standard GB 4789.7–2013 method was used as a reference assay to ensure that the food samples were successfully contaminated. The RPA and real-time RPA assays rendered a high degree of agreement (100%) with the real-time PCR results. However, real-time RPA had an overwhelming advantage in terms of time-savings. *V. parahaemolyticus* could be detected by real-time RPA within 5–12 min in oyster sauce, codfish and sleeve-fish at concentrations as low as 4 CFU/25 g, 1 CFU/25 g and 7 CFU/25 g, respectively, after enrichment for 6 h, but detection using real-time PCR required a minimum of 35 min (Ct values between 27 and 32). The detection time decreased in relation to increased amounts of spiked cells and enrichment time. The shortest time to obtain results by real-time RPA was 2.72 min (Tables 3, 4 and 5). These results strongly suggest that the real-time RPA technique has the distinct advantages of rapidity and sensitivity.

**Table 3:** The comparison of detection results of different methods in contaminated oyster sauce

Spiked cells (CFU/25 g oyster sauce)	Enrichment time (h)	Real-time RPA (min)	Real-time PCR (Ct)	RPA (min)	GB4789.7 (Day)	Viable cell counts (CFU/g)
4	6	5.02	32.48	20	3	4.4 × 10^2^
	8	3.35	21.45	20	3	4.8 × 10^2^
25	6	4.03	27.15	20	3	6.3 × 10^3^
	8	2.88	20.36	20	3	4.1 × 10^3^
80	6	3.72	21.34	20	3	1.0 × 10^4^
	8	2.72	16.32	20	3	1.0 × 10^4^

**Table 4:** The comparison of detection results of different methods in contaminated codfish

Spiked cells (CFU/25 g codfish)	Enrichment time (h)	Real-time RPA (min)	Real-time PCR (Ct)	RPA (min)	GB4789.7 (Day)	Viable cell counts (CFU/g)
1	6	12.02	30.64	20	ND	0
	8	11.03	29.18	20	ND	0
10	6	6.07	25.12	20	3	5.2 × 10^3^
	8	5.37	20.43	20	3	7.1 × 10^3^
42	6	5.07	17.45	20	3	2.2 × 10^4^
	8	4.20	17.15	20	3	7.2 × 10^4^

**Table 5:** The comparison of detection results of different methods in contaminated sleeve-fish

Spiked cells (CFU/25 g sleeve-fish)	Enrichment time (h)	Real-time RPA (min)	Real-time PCR (Ct)	RPA (min)	GB4789.7 (Day)	Viable cell counts (CFU/g)
7	6	8.15	27.05	20	3	8.9 × 10^2^
	8	7.35	21.33	20	3	9.1 × 10^3^
10	6	6.33	25.31	20	3	7.5 × 10^2^
	8	6.03	20.45	20	3	9.5 × 10^3^
56	6	6.05	17.40	20	3	1.0 × 10^5^
	8	5.72	18.53	20	3	3.1 × 10^5^

*ND* Not detected

## Discussion

In recent years, diseases caused by food-borne pathogens have become a significant global public health issue. *V. parahaemolyticus* is one of the major pathogens causing food-borne illness, and contamination of food products with this pathogen has become a vital concern for food safety [9, 28]. Therefore, there is a need for rapid, specific, and reliable diagnostic techniques that can be used effectively for better detection of *V. parahaemolyticus* in seafood, environmental, and other various sample types.

In this report, the real-time RPA assay targeting the *gyrB* gene of *V. parahaemolyticus* was successfully established. Conventional RPA was successfully performed at 38 °C and completed within 20 min. The sensitivity of the real-time RPA assay was examined using serial dilutions of *V. parahaemolyticus* genomic DNA template. The limit of detection of the real-time RPA assay was 10^2^ copies/reaction. While the *V. parahaemolyticus* detection results by real-time RPA could be obtained in approximately 30 min, including the time for nucleic acid extraction, the reaction time for positive samples reached up to 1 h when using real-time PCR [11]. In our study, the real-time RPA assay is an exact match to 99% of the sequences in GenBank. The other *gyrB* target sequences, approximately 1%, were mismatched. Further analysis showed that the primer probes sequence used in this paper with other *Vibrio parahaemolyticus* had at most 3 mismatched bases, usually 1 or 2. As one of advantage, the RPA assay has demonstrated a certain tolerance to a certain length mismatch within RPA primers and exo-probe that do not influence the performance of RPA reactions, generally 3–5 mismatches according to previous research [29, 30]. To verify the specificity of real-time RPA for the detection of *V. parahaemolyticus,* a variety of bacterial strains, listed in Table 1, were tested. The results showed that real-time RPA and real-time PCR technology both had a high degree of specificity to *V. parahaemolyticus*. However, the further sequence optimization and testing is required to ensure the assay efficiently detects all of the targeted species when it is to be applied in practice in the future.

In addition, the real-time RPA assay was also successful in the detection of artificially contaminated seafood samples, and it performed better than real-time PCR with respect to detection speed.

In recent years, a number of isothermal DNA amplification methods have been developed as a simple, rapid technique alternative to PCR-based amplification, which enable the detection of minute amounts of nucleic acid and are ideally suited to field situations [19–21]. Loop-mediated isothermal amplification (LAMP) and the cross-priming amplification assay (CPA) have been adopted for rapid and sensitive detection of *V. parahaemolyticus* in seafood samples [18, 31]*.* In the LAMP assay, a set of four primers is needed, and the optimum time and temperature are 60 min and 65 °C, respectively. Wang et al. established the real-time loop-mediated isothermal amplification technique (MERT-LAMP) for the detection of *V. parahaemolyticus* infection, which overcame the limitations posed by current LAMP technologies and allowed for real-time detection of multiple, distinct targets [16]. However, the optimal MERT-LAMP amplification temperature is 62 °C and reactions require 60 min, which is much longer than the real-time RPA assay we used in this study. CPA was able to detect as low as 1.8 CFU/mL for pure cultures and 18 CFU/g for reconstituted samples within 60 min [18, 19]. For the real-time RPA assay described in this study, *V. parahaemolyticus* could be detected in artificially contaminated samples at concentrations as low as 1 CFU/25 g within 12 min. Compared to other isothermal amplification techniques, RPA does not require initial heating for DNA denaturation, and results can be obtained in less than 12 min which didn’t include the enrichment time. The real-time RPA assay has multiple advantages over other DNA amplification methods, including a quicker time-to-result for a single sample; and the potential for reduced impact of matrix-associated inhibitors [20]. RPA has been widely explored for the molecular detection of diverse pathogens, and field testing has also been achieved for Dengue virus and avian influenza A virus infection [32, 33]. Moreover, the portable POC tube scanner (Genie III, OptiGene Limited, West Sussex, United Kingdom) used in the study, weighing only 1.75 kg with dimensions of 25 cm × 16.5 cm × 8.5 cm, is simpler than most real-time PCR machines and can be used in the field, running on battery power for an entire day.

## Conclusion

In conclusion, the real-time RPA method based on an exo probe was successfully developed for the detection of *V. parahaemolyticus*. With high sensitivity and specificity, the assay could be completed within 20 min and the approach is easy to perform in clinical settings without a requirement for sophisticated equipment, which renders it applicable at quarantine stations, ports or sites of outbreaks. The effective and rapid real-time RPA assay developed in this study would be highly useful in the monitoring of *V. parahaemolyticus* infection and has the potential to be a promising alternative to real-time PCR and other isothermal methods for rapidly testing *V. parahaemolyticus* infection*.*

## Data Availability

The dataset analyzed during the current study is available from the corresponding author on reasonable request.

## Abbreviations

ATCC: American Type Culture Collection
CFU: colony-forming units
CICC: China Center of Industrial Culture Collection
CMCC: China Center for Medical Culture Collection
CPA: cross-priming amplification
Ct: Cycle threshold
FP: Forward primer
LAMP: loop-mediated isothermal amplification
LOD: Limit of detection
PCR: Polymerase Chain Reaction
RP: Reverse primer
RPA: recombinase polymerase amplification
SSB: signal-stranded DNA-binding protein
